# Supplementary material for: The interactome of intact mitochondria by cross-linking mass spectrometry provides evidence for coexisting respiratory supercomplexes
Source: Mol Cell Proteomics. 2017 Dec 8;17(2):216–32. doi: 10.1074/mcp.RA117.000470 (PMC5795388; doi:10.1074/mcp.RA117.000470)
Supplement: Supplemental Data [file supp_RA117.000470_133922_0_supp_23978_fzffwf.zip › spectra_annotation/mito_spectra_annotation/291-1-9-1-10-1.pdf]

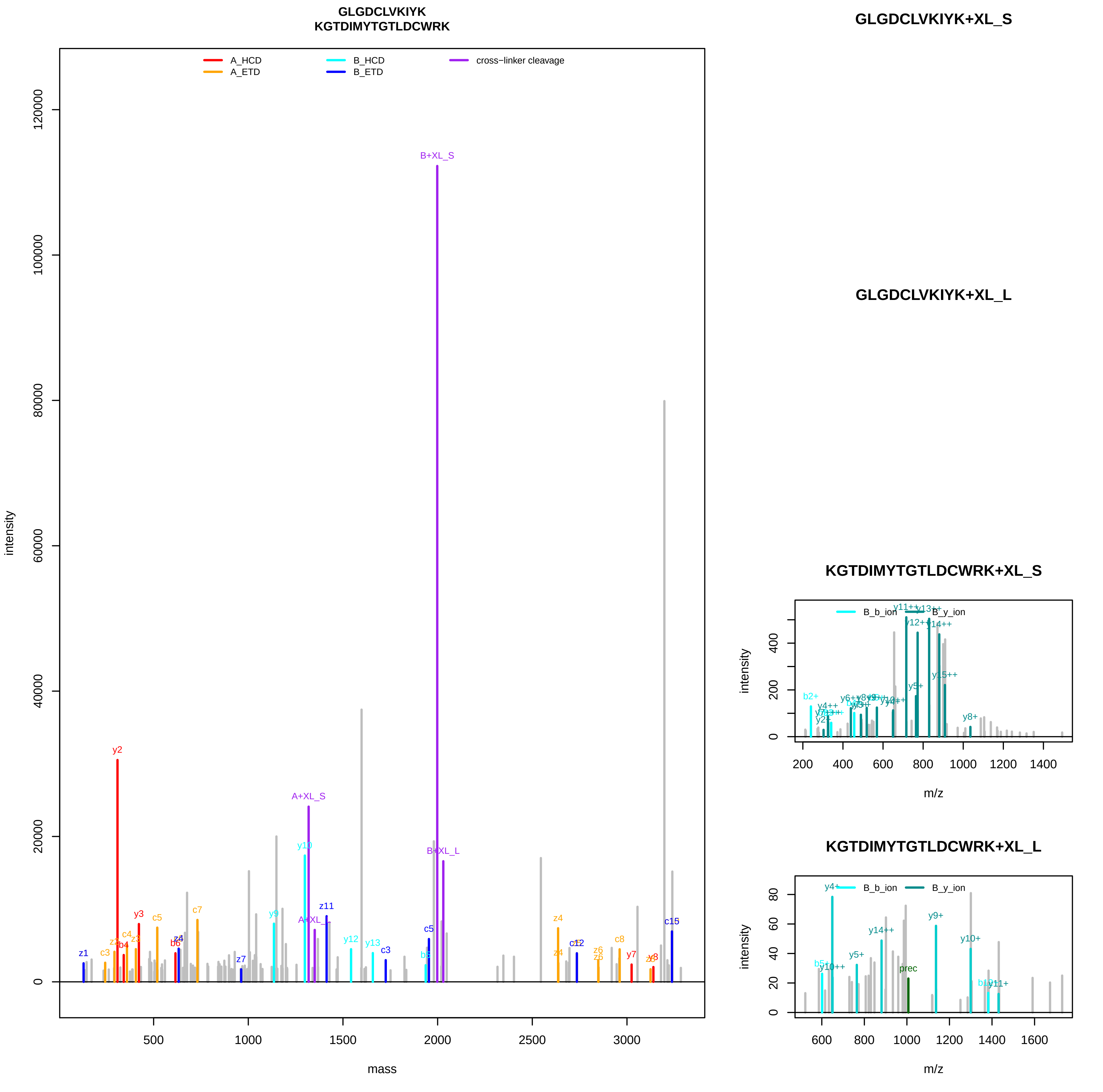

GLGDCLVKIYK
KGTDIMYTGTLDCWRK
A_HCD
A_ETD
B_HCD
B_ETD
cross−linker cleavage
intensity
mass
B+XL_S
A+XL_S
B+XL_L
y2
y3
y7
y8
c5
c7
c8
z4
z6
y10
y12
y13
z11
c15
c12
c3
c5
z7
z1
GLGDCLVKIYK+XL_S
GLGDCLVKIYK+XL_L
KGTDIMYTGTLDCWRK+XL_S
KGTDIMYTGTLDCWRK+XL_L
B_b_ion
B_y_ion
m/z
prec
y9+
y10+
y14++
y4+
y5+
y8+
y15++
y5+
